# RDE Treatment Prevents Non-Specific Detection of SARS-CoV-2- and Influenza-Specific IgG Antibodies in Heat-Inactivated Serum Samples

**DOI:** 10.3390/antib12020039

**Published:** 2023-06-16

**Authors:** Arina Goshina, Victoria Matyushenko, Daria Mezhenskaya, Alexandra Rak, Anastasia Katelnikova, Denis Gusev, Larisa Rudenko, Irina Isakova-Sivak

**Affiliations:** 1 Department of Virology, Institute of Experimental Medicine, 197376 Saint Petersburg, Russia; arina8goshina@gmail.com (A.G.); matyshenko@iemspb.ru (V.M.); dasmez@iemspb.ru (D.M.); alexandrak.bio@gmail.com (A.R.);; 2 Department of Toxicology and Microbiology, Institute of Preclinical Research Ltd., 188663 Saint Petersburg, Russia; katelnikova.ae@doclinika.ru; 3 Botkin Infectious Diseases Hospital, Piskarovskiy Ave 49, 195067 Saint Petersburg, Russia

**Keywords:** SARS-CoV-2, influenza, ELISA, heat inactivation, serum antibody, receptor-destroying enzyme

## Abstract

Assessing the levels of serum IgG antibodies is widely used to measure immunity to influenza and the new severe acute respiratory syndrome coronavirus 2 (SARS-CoV-2) after natural infection or vaccination with specific vaccines, as well as to study immune responses to these viruses in animal models. For safety reasons, sometimes serum specimens collected from infected individuals are subjected to heat inactivation at 56 °C to reduce the risk of infecting personnel during serological studies. However, this procedure may affect the level of virus-specific antibodies, making the results of antibody immunoassays uninterpretable. Here, we evaluated the effect of the heat inactivation of human, ferret and hamster serum samples on the binding of IgG antibodies to the influenza and SARS-CoV-2 antigens. For this, serum samples of naive and immune hosts were analyzed in three variants: (i) untreated sera, (ii) heated at 56 °C for 1 h, and (iii) treated with receptor-destroying enzyme (RDE). The samples were studied through an in-house enzyme-linked immunosorbent assay (ELISA) using whole influenza virus or recombinant proteins corresponding to nucleocapsid (N) protein and the receptor-binding domain of SARS-CoV-2 Spike (RBD) as antigens. We demonstrated that the heat inactivation of the naive serum samples of various hosts can lead to false-positive results, while RDE treatment abolished the effect of the non-specific binding of IgG antibodies to the viral antigens. Furthermore, RDE also significantly decreased the level of virus-specific IgG antibodies in SARS-CoV-2 and influenza-immune sera of humans and animals, although it is unknown whether it actually removes true virus-specific IgG antibodies or only non-specifically binding artifacts. Nevertheless, we suggest that the RDE treatment of human and animal sera may be useful in preventing false-positive results in various immunoassays, while also neutralizing infectious virus, since the standard protocol for the use of RDE also includes heating the sample at 56 °C.

## 1. Introduction

The new coronavirus disease pandemic (COVID-19), which began in 2020, has led to a severe socioeconomic crisis worldwide, and SARS-CoV-2 is likely to be added to the list of viruses that cause seasonal respiratory infections in humans [1,2]. Coronaviruses affect many different species of animals and humans, where they may cause mild or severe respiratory infections [3]. Vaccines against SARS-CoV-2 have been developed at an unprecedented rate, significantly slowing the spread of infection, reducing the risk of long COVID and saving millions of lives [4,5,6]. However, the constant emergence of new strains of SARS-CoV-2, partially because of the massive implementation of spike-based vaccines, continues to be a significant burden for global public health [7,8,9]. Therefore, attempts to develop a new generation of COVID-19 vaccines targeting less mutation-prone viral antigens are continuing using various vaccine platforms [10,11].

The human immune responses to natural SARS-CoV-2 infection and vaccination have been extensively studied, and a wealth of data have been accumulated concerning the magnitude and duration of humoral immunity, as well as T-cell immune responses [12,13,14,15]. When analyzing virus-specific immunity developed to natural infection and vaccination, most researchers focus on the evaluation of SARS-CoV-2-neutralizing antibodies, since they play a crucial role in preventing viral entry into the host cell and therefore correlating with protection against reinfection [16,17,18]. Additionally, non-neutralizing antibodies induced by coronavirus infection or vaccination can mediate additional immune functions that may have both protective and pathological consequences [19]. Thus, non-neutralizing and weakly neutralizing antibodies induced by severe acute respiratory syndrome coronavirus (SARS-CoV) and Middle East respiratory syndrome coronavirus (MERS-CoV) vaccines caused disease progression, a phenomenon known as the antibody-dependent enhancement of infection (ADE) [20,21]. Similarly, some non-neutralizing antibodies targeting the conserved stem domain of hemagglutinin (HA) of influenza virus can provoke disease enhancement, where a possible mechanism is an increase in influenza virus fusion kinetics via the destabilization of HA stem domain [22,23]. Hence, it is important to assess all antibody subsets that can bind with the virus, regardless of their neutralizing capacity. There are several types of immunoassays that can assess virus-specific humoral immunity, such as enzyme-linked immunosorbent assay (ELISA), chemiluminescent immunoassays (CLIAs) and lateral flow immunoassays (LFIAs), where any desired antigenic determinants can be used as coating antigens. ELISA/CLIA-based methods are generally more sensitive than LFIA [24], while ELISA is less expensive than CLIA and is most commonly used in routine research of immune responses to viral pathogens using animal models.

In studies of humoral immune responses to SARS-CoV-2, human and animal sera are considered potentially infectious material; therefore, the thermal inactivation of samples at 56 °C for 1 h is recommended to reduce the risk of infection of personnel [25]. It is known that heat inactivation may have significant consequences for the results of some immunoassays. For example, serum heat inactivation has been shown to increase the specificity of the competitive Luminex Immunoassay for human papilloma virus (HPV), because some heat-labile serum factors interfered with the binding of detection monoclonal antibodies to the HPV antigens, leading to false-positive results [26]. In the case of the West Nile virus, the heat inactivation of immune mouse sera increased the sensitivity of the Microsphere immunoassay by eliminating complement interference, thereby facilitating the detection of antibodies in the early and late phases of infection [27]. For some other viruses, in contrast to this, the heating of sera at 56 °C can lead to false-positive results. This phenomenon was described for human T-cell lymphotropic virus type III [28] and, more recently, for SARS-CoV-2 [29,30,31]. Given that the thermal inactivation of serum samples from animals used in preclinical trials of SARS-CoV-2 vaccines may also be required for safety reasons, it is important to evaluate the effect of such a treatment on the results of serological testing. Specifically, ferrets and hamsters are the most widely used models for studying virus-specific immune responses and preclinical trials of vaccines against SARS-CoV-2 and influenza because they are naturally susceptible to both infections and recapitulate many clinical signs of either disease [32,33,34].

Here, we evaluated the effect of the thermal inactivation of human, ferret and hamster sera on the binding of IgG antibodies to the nucleocapsid protein (N) and to the receptor-binding domain (RBD) of SARS-CoV-2 Spike protein, which are the most widely used antigens in SARS-CoV-2 serology tests [35,36]. Similarly, we studied the effect of heat inactivation on the detection of influenza whole virus-specific antibodies, and both naïve and immune serum samples were used in the analyses. In addition, serum treatment with a receptor-destroying enzyme (RDE) was performed to determine whether the non-specific binding of serum proteins with viral antigens can be eliminated to avoid false-positive results. RDE, which is a sialidase from *Vibrio cholerae*, is routinely used to inactivate sialylated innate inhibitors of influenza virus when conducting hemagglutination inhibition assay (HAI) [37], as well as to prevent the non-specific, non-antibody-mediated inhibition of influenza virus replication in a microneutralization assay [38]. Whether RDE treatment can affect antibody binding with SARS-CoV-2 or influenza antigens has not been previously studied.

## 2. Materials and Methods

### 2.1. Viruses and Proteins

Three reassortant influenza viruses for live attenuated influenza vaccine (LAIV) were used as coating antigens for influenza-based ELISAs in this study:

1. H3N2 LAIV: a 6:2 reassortant virus on the A/Leningrad/17 backbone bearing two genes coding for hemagglutinin (HA) and neuraminidase (NA) of the A/Switzerland/9715293/2013 (H3N2) virus;
2. H5N2 LAIV: a Len/17-based LAIV with a genome formula of 7:1, where only the HA gene was inherited from avian influenza virus A/turkey/Turkey/1/2005 (H5N1), while the remaining seven genes originate from the Len/17 MDV;
3. H7N9 LAIV: a Len/17-based LAIV strain expressing HA and NA genes of a potentially pandemic A/Anhui/1/2013 (H7N9) virus.

The influenza viruses were grown in 10–12-day-old embryonated chicken eggs. To prepare viral antigens for ELISA, the viruses were purified on a sucrose cushion using the following procedure: Pooled harvested allantoic fluid was clarified using low-speed centrifugation (3500 rpm, 15 min), followed by viral sedimentation at 19,000 rpm for 3 h at 4 °C using an ultracentrifuge (Beckman Coulter, Brea, CA, USA). Viral precipitate was resuspended in 1 mL of sterile phosphate-buffered saline (PBS) and layered on top of 30%/60% sucrose step gradient, followed by centrifugation at 23,000 rpm for 2 h at 4 °C. The interphase was collected and washed in PBS through additional centrifugation at 23,000 rpm for 1 h at 4 °C. The final precipitate was resuspended in 1 mL of PBS and stored in aliquots at −70 °C.

A recombinant N protein of SARS-CoV-2 isolate hCoV-19/St_Petersburg-3524S/2020 (GISAID EPI_ISL_415710, Munich, Germany) was generated earlier and its identity was confirmed using mass spectrometric analysis [39,40].

A recombinant RBD protein (amino acid residues 319–541) was produced by Joint Stock Company BIOCAD (St. Petersburg, Russia) [41].

### 2.2. Serum Samples

#### 2.2.1. Humans

Twenty serum samples positive for SARS-CoV-2 IgG antibodies were collected from 20 COVID-19 convalescents in 2020 and 2021. Blood donors were recruited during the study of SARS-CoV-2-specific humoral and T-cell responses after COVID-19 [42], which was approved by the Ethics Committee of the Institute of Experimental Medicine (protocol №2/20 dated 7 April 2020). Study participants were aged 31 to 77 years, and the disease onset ranged between May 2020 and March 2021, and time of post-symptom onset (PSO) was from 1 to 12 months. All patients signed an informed consent form.

Serum samples positive for influenza H5N1 IgG antibodies (*n* = 10), as well as control historical serum samples that were negative both for SARS-CoV-2 and influenza H5N1 IgG antibodies (*n* = 20), were taken from archived specimens collected from healthy adults participated in Phase I trial of H5N2 LAIV (NCT01719783) [43] and in the study of immunogenicity of H5N1-inactivated influenza vaccine following H5N2 LAIV (NCT02153671) [44].

#### 2.2.2. Ferrets

SARS-CoV-2 IgG-positive ferret serum samples (*n* = 20) were obtained from the Institute of Preclinical Research (Leningrad region, Russia). The ferrets tested positive during a screening procedure within a study of safety and immunogenicity of a COVID-19 vaccine candidate (the study was approved by the Local Ethical Committee of the Institute of Preclinical Research No. BEC 2.40/21 dated 11 August 2021). Ten serum specimens collected from ferrets immunized with H3N2 LAIVs [45] were used as samples positive for influenza H3N2 IgG antibodies. The control group included serum samples from 20 naïve animals collected as part of preclinical trials of H7N9 LAIVs [46].

#### 2.2.3. Hamsters

Ten serum samples of SARS-CoV-2 IgG-positive hamsters, as well as 10 control samples from naïve animals, were collected during a study in which a new test system was developed to evaluate the cell-mediated immune response to SARS-CoV-2 in Syrian hamsters [47]. Influenza IgG-positive serum samples were taken from an immunogenicity and protection study of a bivalent vaccine against SARS-CoV-2 and influenza virus, which was developed on the H7N9 LAIV virus vector [48].

All serum samples were stored in aliquots at −20 °C prior to analysis.

### 2.3. Treatment of the Serum Samples

Sera from all hosts were analyzed in three variants: (i) untreated specimens; (ii) heat-inactivated specimens (56 °C); and (iii) RDE-treated specimens.

For heat inactivation, aliquots of undiluted sera were incubated at 56 °C for 1 h in a water bath, followed by cooling at 4 °C prior to the use in ELISA. Treatment of serum samples with RDE (Denka Seiken, Tokyo, Japan) was performed according to the instruction of the manufacturer. Briefly, one volume of a serum sample was added to three volumes of RDE and incubated for 24 h at 37 °C. Then, the serum was incubated in a water bath at 56 °C for 30 min to inactivate the RDE, followed by cooling at room temperature and adjusting to a final dilution of 1:10 by adding six volumes of PBS.

### 2.4. Assessment of Serum IgG Antibody Levels in ELISA

Serum IgG antibody levels to SARS-CoV-2 antigens and whole influenza viruses were assessed by in-house ELISA. Briefly, 96-well MaxiSorp plates (ThermoFisher Scientific, Waltham, MA, USA) were coated with SARS-CoV-2 recombinant N protein or RBD protein at a concentration of 100 ng per well. The sucrose gradient-purified whole influenza viruses were sorbed at 16 hemagglutinating units per well. All antigens were diluted in a carbonate-bicarbonate buffer (pH 9.7) and the coated plates were incubated overnight at 4 °C. After blocking with 1% bovine serum albumin (BSA) in PBS, the wells were washed three times with washing buffer (PBS with 0.05% Tween 20, PBST). Then, 50 µL of 2-fold dilutions of sera were added to the wells. Naïve serum samples were diluted starting from 1:10, whereas immune sera were diluted starting from 1:100. After incubation for 1 h at 37 °C and washing, 50 μL of horseradish peroxidase-conjugated species-specific secondary antibodies in the dilution indicated by the manufacturer (Sigma, Burlington, MA, USA) were added to the wells and incubated at 37 °C for 1 h. The plates were then washed 5 times with PBST and 50 μL of 1-Step Ultra TMB-ELISA substrate solution (Thermo, Waltham, MA, USA) was added. After 15 min incubation, the reaction was stopped by adding 25 μL of 1M H_2_SO_4_, and the results were read at 450 nm with an xMark Microplate spectrophotometer (BioRad, Hercules, CA, USA). Individual optical density (OD) values from selected dilutions (1:40 for naïve sera and 1:400 for immune sera) were used for statistical calculations.

### 2.5. Statistical Analysis

The results were analyzed in GraphPad Prism software version 8.0 (GraphPad Software, San Diego, CA, USA) using a non-parametric Wilcoxon Signed Rank test for pairwise comparison of the three groups of sera (untreated, heat-inactivated at 56 °C and RDE-treated). *p* values of <0.05 were considered significant.

## 3. Results

In this study, we evaluated the potential effect of the thermal inactivation of human, ferret and hamster sera on the detection of SARS-CoV-2-specific and influenza-specific antibodies by ELISA.

### 3.1. Effect of Heat Inactivation and RDE Treatment on the Levels of SARS-CoV-2-Specific IgG Antibody

**Figure 1:**
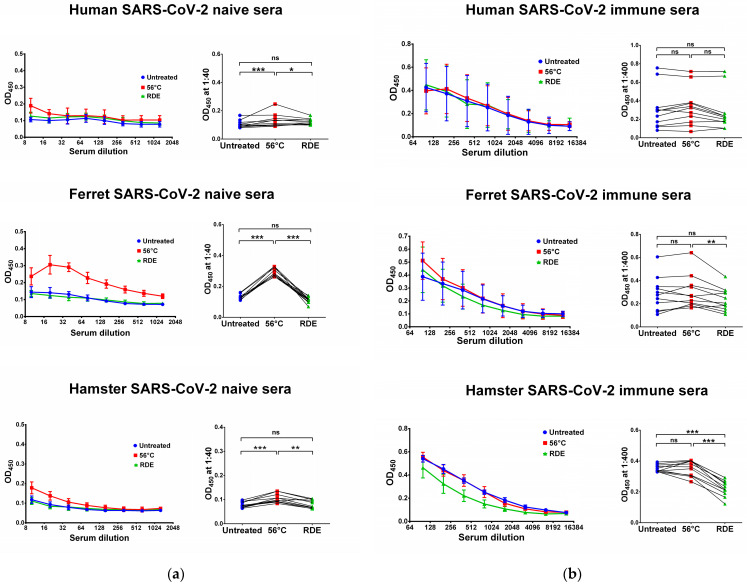
Serum IgG antibodies in SARS-CoV-2-naïve (**a**) and -immune (**b**) specimens detected in ELISA against SARS-CoV-2 N antigen. Upper panel shows human serum specimens (*n* = 11). Middle panel shows ferret serum specimens (*n* = 11). Lower panel shows hamster serum specimens (*n* = 11). Left panels for each graph show the mean OD_450_ values for each serum dilution. Right panels show the OD_450_ values for each specimen at a dilution of 1:40 (for naïve samples) or 1:400 (for immune samples). The data were analyzed with Wilcoxon Signed Rank test for pairwise comparison. * *p* < 0.05; ** *p* < 0.01; *** *p* < 0.001. ns, not significant.

**Figure 2:**
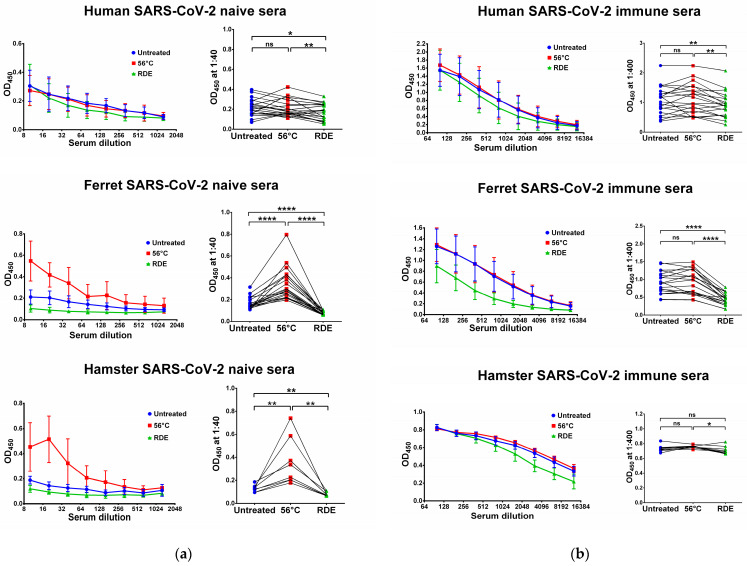
Serum IgG antibodies in SARS-CoV-2-naïve (**a**) and -immune (**b**) specimens detected in ELISA against SARS-CoV-2 RBD antigen. Upper panel shows human serum specimens (*n* = 20). Middle panel shows ferret serum specimens (*n* = 20). Lower panel shows hamster serum specimens (*n* = 10). Left panels for each graph show the mean OD_450_ values for each serum dilution. Right panels show the OD_450_ values for each specimen at a dilution of 1:40 (for naïve samples) or 1:400 (for immune samples). The data were analyzed with Wilcoxon Signed Rank test for pairwise comparison. * *p* < 0.05; ** *p* < 0.01; **** *p* < 0.0001. ns, not significant.

We used two SARS-CoV-2 recombinant proteins as antigens for in-house ELISA, namely N and RBD proteins. Untreated serum samples of humans, ferrets and hamsters who had no history of contact with SARS-CoV-2 infection did not bind with the N (Figure 1a) and RBD (Figure 2a) antigens. Interestingly, the heat inactivation of the sera at 56 °C significantly increased the levels of antigen-binding IgG antibodies in all cases, with the exception of RBD-binding human specimens (Figure 2a). This effect of non-specific IgG antibody binding was the most pronounced for animal sera, where much higher OD_450_ values were seen in the heat-inactivated groups compared to the untreated specimens, both for N and RBD antigens (Figure 1a and Figure 2a). These data indicate that the heat treatment of sera obtained from non-immune ferrets and hamsters can lead to false-positive IgG ELISA results. Strikingly, the treatment of the same serum specimens with RDE completely abolished the ability of the naïve sera to bind with SARS-CoV-2 antigens (Figure 1a and Figure 2a). When RDE-treated sera were compared to the untreated samples, there was no difference in the N-specific IgG antibody levels (Figure 1a). Although RDE treatment statistically significantly reduced non-specific binding to the RBD antigen for all species tested (Figure 2a), these differences cannot be considered clinically significant because of the very low antibody levels.

We further assessed serum IgG antibody levels in samples of COVID-19 convalescents and SARS-CoV-2-infected animals. The recovered COVID-19 patients had diverse N- and RBD-specific serum IgG antibody levels, since they vary significantly in disease severity and the time of post-symptom onset (Figure 1b and Figure 2b). Similarly, ferrets were heterogeneous in their seropositivity for SARS-CoV-2, most likely due to differences in their infection history, which cannot be traced because ferrets can be infected asymptomatically [49,50]. In contrast, hamsters were inoculated with the same dose of SARS-CoV-2, and serum samples were collected at one time point, thus producing more homogenous IgG antibody levels (Figure 1b and Figure 2b).

Importantly, the heat inactivation of SARS-CoV-2-immune sera did not increase antibody binding to N and RBD antigens for all species studied, suggesting that the non-specific binding of serum components after heating does not make any significant contribution to the overall pool of binding antibodies. Quite unexpectedly, antibody binding to RBD protein was significantly reduced when immune human and ferret sera were treated with RDE (Figure 2b), whereas this effect was not seen for the N antigen (Figure 1b). Hamster serum samples had the same effect for N SARS-CoV-2 antigen, but not for RBD antigen (Figure 1b and Figure 2b).

### 3.2. Effect of Heat Inactivation and RDE Treatment on the Levels of Influenza-Specific IgG Antibody

**Figure 3:**
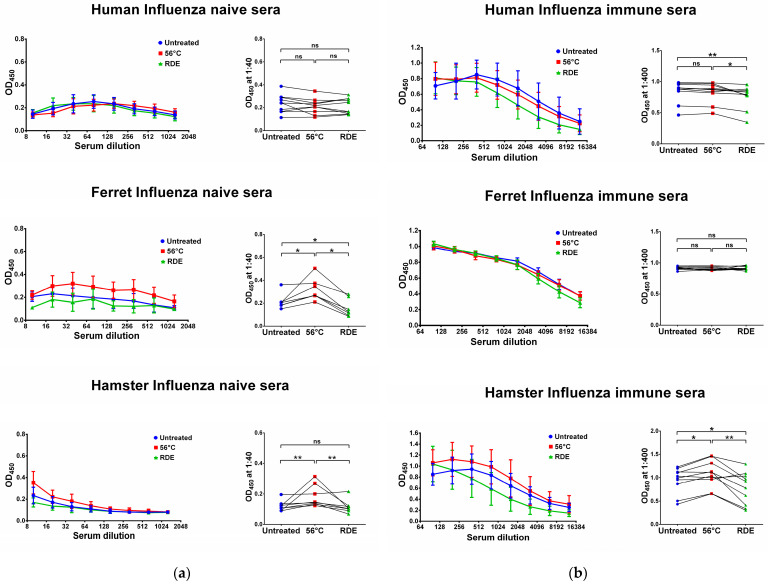
Serum IgG antibodies in influenza-naïve (**a**) and -immune (**b**) specimens detected in ELISA against whole influenza virus antigens. Upper panel shows human serum reactivity to H5N1 influenza virus (n = 10). Middle panel shows ferret serum reactivity to H3N2 influenza virus (n = 7). Lower panel shows hamster serum reactivity to H7N9 influenza virus (n = 10). Left panels for each graph show the mean OD_450_ values for each serum dilution. Right panels show the OD_450_ values for each specimen at a dilution of 1:40 (for naïve samples) or 1:400 (for immune samples). The data were analyzed with Wilcoxon Signed Rank test for pairwise comparison. * *p* < 0.05; ** *p* < 0.01. ns, not significant.

We further assessed whether the same effect of serum heat inactivation can be seen when analyzing influenza-specific antibody responses in naïve and immune sera of humans, ferrets and hamsters. We used archived human serum specimens from a study of immune responses to H5N1-inactivated influenza vaccine among individuals previously primed with H5N2 live attenuated influenza vaccine [44]. Since H5N1 avian influenza viruses have not circulated in the human population, serum specimens collected prior to the immunization with H5N2 LAIV were considered influenza-naïve in our study. Sera from subjects who responded to the H5N1 booster immunization were considered as influenza-immune specimens. Unlike SARS-CoV-2 antigens, the reactivity of influenza-naïve and -immune human sera with the whole influenza virus antigen was not affected by serum heating at 56 °C (Figure 3), although the RDE treatment of influenza-immune sera slightly reduced the levels of detectable virus-specific IgG antibodies (Figure 3b).

The reactivity of influenza-immune ferret sera with the homologous antigen was not affected by either RDE treatment or by serum heating at 56 °C (Figure 3b). In contrast, the reactivity of influenza-naïve ferret sera was affected by heating and RDE treatment, where thermal inactivation increased virus-specific IgG antibody levels, whereas RDE treatment significantly reduced antibody binding even compared to the untreated samples (Figure 3a). These data recapitulate the results from the assessment of SARS-CoV-2 antigen-binding IgG antibodies in naïve ferrets after serum treatment (Figure 1a and Figure 2a).

Similar to SARS-CoV-2 antigens, influenza virus-binding IgG antibodies were significantly increased in influenza-naïve hamsters after serum heating at 56 °C (Figure 3a), suggesting the appearance of some factors that non-specifically bind with virus antigens and can eventually lead to false-positive antibody test results. Interestingly, heat inactivation also increased antibody binding in influenza-immune hamsters (Figure 3b), although this effect was not noted in SARS-CoV-2-immune animals (Figure 1b and Figure 2b). Nevertheless, in both SARS-CoV-2 and influenza-immune hamsters, the RDE treatment of serum samples significantly decreased IgG antibody binding with virus antigens in ELISA (Figure 1b, Figure 2b and Figure 3b).

Overall, the most pronounced effect of serum heating at 56 °C was noticed when analyzing naïve serum specimens: this procedure significantly increased sera reactivity to both SARS-CoV-2 and influenza virus antigens, which can have serious diagnostic consequences due to the false-positive test results. Importantly, in most cases serum treatment with the receptor-destroying enzyme can abolish the effect of the non-specific antigen binding of serum IgG antibodies for both tested viruses. Therefore, RDE pretreatment should be considered one of the necessary steps to prepare serum for assays in preclinical and clinical studies where potentially infectious serum samples can be obtained, thus minimizing the contact of personnel with pathogens and reducing the risk of detecting non-specific antibodies. It should be noted that the appearance of non-specific reactivity of sera after heat inactivation was most prominent in animal serum samples than in human sera, which further indicates the importance of proper serum handling in preclinical trials of SARS-CoV-2 and influenza vaccines and in the study of the pathogenicity and immunogenicity of wild-type pathogens in these animals.

## 4. Discussion

The evaluation of serum antibody levels to SARS-CoV-2 is an important tool for diagnosing the humoral immune response in people who have recovered from COVID-19 or have been vaccinated against the disease. There are multiple commercially available SARS-CoV-2 antibody assays with relatively high sensitivities and specificities [51,52] and the most affordable and cost-effective test systems are generally based on an ELISA approach, where N or Spike/RBD proteins of SARS-CoV-2 are used as coating antigens [35,36]. Since biological samples taken from COVID-19 patients are considered potentially infectious, safety standards require the thermal inactivation of the sera by incubating the samples at 56 °C before serological tests. For the majority of viral pathogens, the heating of sera is not expected to have any effect on antibody titers. However, for SARS-CoV-2 infection, significant changes in IgG and IgM antibody levels have been shown in the sera of exposed individuals before and after heat inactivation. For example, a study by Hu et al. [31] demonstrated a significant increase in IgG and IgM antibody levels after the heating of serum samples of COVID-19 patients at 56 °C for 30 min, although this treatment did not affect the diagnostic accuracy of the commercial RBD-based ELISA immunoassay used in this study. In another study, the treatment of human serum specimens at 56 °C for 30 min had a negligible effect on the results of IgG detection using a commercial ELISA test, although a decrease in neutralizing antibody titers was noted [30]. These data are consistent with the results of our study, where heating at 56 °C for 1 h of SARS-CoV-2 immune human sera did not affect the detection of IgG antibodies to both N and RBD protein antigens. However, a significant increase in N-binding antibody levels was noted when naïve SARS-CoV-2 samples were heat-inactivated, suggesting that N-based ELISA immunoassays should be carefully monitored for the appearance of false-positive antibody test results when serum samples are subjected to heat inactivation.

It is well-known that the influence of the complement system often has a significant negative effect on many analytical methods that require working with cell cultures. The heat treatment of sera is commonly used to inactivate the complement system, but it has been shown that IgG antibody aggregates can appear and significantly increase their concentration, and interestingly, this correlates with the albumin concentration in the serum; since the amount of albumin varies greatly from person to person, the formation of IgG antibody aggregates can also vary significantly [53].

To date, there have been no studies of the effects of the heat inactivation of serum samples collected from animals used in studies of the biological properties of SARS-CoV-2 and preclinical trials of the COVID-19 vaccine. Since ferrets and hamsters are two widely used animal models for studying SARS-CoV-2 infection, we examined how the binding of IgG antibodies to virus antigens would change upon the thermal treatment of naive and immune serum samples. Notably, for both species of animals that had never been infected with SARS-CoV-2, there was a significant increase in OD_450_ values when ELISA was performed using thermally inactivated samples compared to the untreated samples. The main reason for the appearance of such “artifacts” may be the structural rearrangement of IgG antibodies and the formation of immunoglobulin aggregates affecting the avidity of the antigen–antibody complex [54]. The presence of disulfide bonds in the native conformation of serum proteins ensures their thermodynamic stability, but these bonds may be broken during prolonged heating due to the disulfide–thiol exchange reaction, which further leads to protein denaturation [55]. Furthermore, a comprehensive analysis of the effects of the thermal treatment of human blood plasma using quantitative nuclear magnetic resonance spectroscopy revealed remarkable changes in the profiles of lipoproteins and low-molecular-weight metabolites, which in turn can lead to the appearance of numerous artifactual pseudomarkers in control individuals and the degradation of real biomolecules SARS-CoV-2-infected patients [56].

It is important to note that a similar phenomenon of the non-specific binding of sera of control ferrets and hamsters during their heat treatment was observed in our study in ELISA with influenza virus antigens. Interestingly, an early study of the humoral immune response to influenza virus in various animal models (horses, guinea pigs, rabbits, and pigs) revealed non-specific inhibitors in serum, which are heat-stable sialoglycoproteins (such as α-2-macroglobulin) competing with specific antibodies for binding and neutralizing the influenza virus [57]. For this reason, measuring influenza-specific antibodies in hemagglutination inhibition assay (HAI) requires the pre-treatment of serum samples with RDE. HAI assay is based on the ability of viral antigenic proteins, such as influenza virus hemagglutinin, to bind red blood cells and induce agglutination. The use of RDE is necessary to hydrolyze serum sialose-containing compounds that can bind to HAs and interfere with the detection of virus-specific antibodies.

In the present study, we investigated whether the use of RDE treatment in immunoassays with SARS-CoV-2 and influenza virus antigens could also affect the detection of specific IgG antibodies to the viral antigens in human and animal sera. Quite unexpectedly, RDE treatment not only abolished the effect of thermal inactivation of naive sera, but also significantly decreased the level of virus-specific IgG antibodies in SARS-CoV-2 and influenza immune sera of humans and animals. It is yet to be understood whether the RDE treatment actually removes true virus-specific IgG antibodies, or only non-specifically binding artifacts are removed from the immune serum. Nevertheless, we suggest that RDE treatment of human and animal sera may be useful in preventing false-positive results in various immunoassays, while also neutralizing infectious virus, since the standard protocol also includes a heating of the sample at 56 °C.

A major limitation of this study is that the results of our in-house ELISAs of human serum specimens were not compared to the commercial SARS-CoV-2 or influenza serology tests, and no conclusions can be drawn about the level of false-positive results in heat-inactivated samples that would be detected in routine large-scale serological screening. However, in addition to human sera, our study included samples from the most relevant animal models that are widely used for testing SARS-CoV-2 and influenza vaccines and antivirals. We acknowledge another limitation of this study, which is the small number of serum specimens included in the analysis; this is due to the fact that we only used archived sera, without involving new animals. However, despite the relatively low sample size, statistically significant differences were found between test groups, suggesting that the observed effects of heat inactivation and RDE treatment may indeed be significant in testing animal serum samples for antibodies to these viruses. Additionally, the results of our study may be of particular importance in experiments where all work with live pathogenic viruses must be handled under BSL-3 conditions, and further assessments of collected samples will be performed in BSL-2 laboratory using a variety of serological assays and different viral antigens.

## 5. Conclusions

In this study, we demonstrated that the heat inactivation of the naive serum samples of various hosts can lead to the false-positive detection of SARS-CoV-2- and influenza-specific antibodies, while RDE treatment abolished the effect of non-specific binding of IgG antibodies to the viral antigens. Although our results do not provide exact cutoff values for deciding whether a sample is positive or negative, based on the differences in OD values observed in this study, it is recommended that the developers of commercial immunoassays should be cautious when interpreting results if a thermal inactivation step of the samples is added to the protocol to increase the safety of personnel.

## Data Availability

The data presented in this study are available on request from the corresponding author.
